# Combining Kidney Organoids and Genome Editing Technologies for a Better Understanding of Physiopathological Mechanisms of Renal Diseases: State of the Art

**DOI:** 10.3389/fmed.2020.00010

**Published:** 2020-02-04

**Authors:** Clara Steichen, Sébastien Giraud, Thierry Hauet

**Affiliations:** ^1^INSERM U1082-IRTOMIT, Poitiers, France; ^2^Université de Poitiers, Faculté de Médecine et de Pharmacie, Poitiers, France; ^3^CHU Poitiers, Service de Biochimie, Poitiers, France

**Keywords:** kidney organoids, genome editing, CRISPR/Cas9, iPSCs, disease modeling, differentiation

## Abstract

Kidney organoids derived from pluripotent stem cells became a real alternative to the use of *in vitro* cellular models or *in vivo* animal models. Indeed, the comprehension of the key steps involved during kidney embryonic development led to the establishment of protocols enabling the differentiation of pluripotent stem cells into highly complex and organized structures, composed of various renal cell types. These organoids are linked with one major application based on iPSC technology advantage: the possibility to control iPSC genome, by selecting patients with specific disease or by genome editing tools such as CRISPR/Cas9 system. This allows the generation of kidney organoïds which recapitulate important physiopathological mechanisms such as cyst formation in renal polycystic disease for example. This review will focus on studies combining these both cutting edge technologies i.e., kidney organoid differentiation and genome editing and will describe what are the main advances performed in the comprehension of physiopathological mechanisms of renal diseases, as well as discuss remaining technical barriers and perspectives in the field.

## Introduction

In nephrology, all commonly used models in fundamental and translational research have their own limits: *in vitro* models are restricted to one cell type analysis without consideration of the intercellular and environmental interactions within the tissue. Animal models, from the isolated organ to the whole organism, are integrative, physiological, and predictive but each individual from an experimental group can react differently to the same treatment, leading to non-negligible bias, in addition to high time and cost aspects. Moreover, ethical laws are limiting the use of living animals for scientific purposes. Evolution toward one intermediary model is a necessity; kidney organoids derived from pluripotent stem cells are one judicious alternative to this issue.

## Kidney Development and Renal Progenitor Cells

Both precursor tissues of the kidney, the ureteral bud (UB, epithelial structure), and the metanephric mesenchyme (MM, mesenchymal tissue) are deriving from intermediate mesoderm. Reciprocal inductive interactions between these both structures lead to the formation of the metanephros, the mature structure of the mammal kidney (1). Indeed, signals coming from MM cells, including the Glial-derived neurotrophic factor, induce UB formation from the Wolffian duct. UB invade the MM secreting notably WNT9b, attracting cells from the MM which condense, forming the cap mesenchyme (CM). The latter is the niche where are NPCs (Nephron Progenitor Cells). These cells specifically express Osr1, Pax2, Six1, Six2 which are necessary for their self-renewal, as well as Vimentin, one mesenchymal marker. Responding to UB signal, these cells secrete Wnt4 which act in an autocrine manner and induce cell epithelialization: this mesenchymal-epithelial transition (MET) being accompanied by E-cadherin expression, while specific genes from undifferentiated cells are switched off. Those epithelial cells are aggregating into vesicles which form nephrons, from the glomeruli to the distal tubule. Cells forming collecting duct and ureter are cells deriving from UB and connect kidney to bladder.

Because of their potential to give rise to the whole tubule, having access to the progenitor cells hosted in the CM would be of great interest for regenerative medicine. But NPC can hardly persist outside their developmental niche. Few days after mouse birth and at 34 weeks of pregnancy in humans, NPCs are fully differentiated (2); thus, formation of new nephrons within the adult kidney does not exist, explaining at least partially why the kidney, despite some evidences of post-lesions regeneration process (3), is not able to adapt and deal with numerous physiopathological situations affecting it.

From fetal kidney tissue, it has been reported the isolation and culture of rodent NPCs (4), but these cells can be cultivated for few passages only (4). The development of specific culture media mimicking NPC niche *in situ* allowed the culture of murine NPCs for up to 10 passages (5). Moreover, using 3 dimensions culture techniques allowing to consider and mimic the physical environment of cells within the kidney, Li et al. showed the possibility to extend murine and human NPC culture from up to 110 and 50 passages, respectively (6).

## Toward Kidney Organoids

The first renal organoids were obtained by relying on an inherent property of NPCs: placed under non-adherent conditions, in 3 dimensions, these cells aggregate, organize themselves spontaneously, forming a cluster of nephrons, and collecting tubes (7). Although the structures obtained are not organized similarly to a kidney, the term organoid can be employed since one possible definition of this term is: three-dimensional structure, organ-like, self-assembled *in vitro* from specific progenitors (8).

More recent studies show the generation of structures with a higher level of organization, with nephrons in a distinct cortex, connected to collecting tubes radiating from the medullary and Henle handles plunging from the cortex into the medullary (9, 10). In such structures, nephrons may exhibit physiological activity (11) though the absence of a ureter naturally prevents the evaluation of all renal functions.

In order to mimic spatial gradients existing in the *in situ* kidney development, placing in contact one side of the organoid with beads releasing BMP4 (bone morphogenetic protein) induces the geo-specific differentiation of cells that are close to it toward “ureter-like” cells of the collecting tube, allowing to break the symmetry of the organoid by raising its anatomical realism (12).

## Pluripotent Stem Cell Derived Kidney Organoids

For years limited to animal models, the study of embryonic development in humans has become possible with the isolation and subsequent culture of embryonic stem cells. These pluripotent cells have the ability to differentiate into all cell types of an adult organism (13). Less than 10 years after this work, the possibility of inducing somatic cells to pluripotency by cellular reprogramming has extended the field of possibilities: iPSCs (induced Pluripotent Stem Cells) technology, in addition to avoiding the use of a human embryo, allows to select the genetic background of cells since they can be derived from an individual with a specific genetic disease for instance (14).

Injected into immunodeficient mice, pluripotent stem cells (PSCs) form teratomas, tumors composed of tissues from the 3 embryonic layers. These teratomas can include renal tubules and glomeruli, showing the ability of PSCs to differentiate into renal tissue *in vivo* (13). But *in vivo* differentiation under these conditions is not specific; *in vitro*, generating kidney cells from PSCs is possible, but the complexity lies in directing this differentiation specifically toward one cell type by controlling the culture conditions; in order to tend toward an efficient and reproducible protocol.

Regarding kidney, the first step of a differentiation protocol consists in differentiating the PSCs into cells of the primitive streak. This is achieved by activating the Wnt, Activine/Nodal, and BMP4 signaling pathways. Specifically, the Activine/BMP4 balance allows the induction of either the anterior or posterior primitive line. A BMP4 (high concentration) + Activin (low concentration) dosage allows to direct rather toward the posterior primitive line, that includes the paraxial and intermediate mesoderm, the latter being the one of interest for the kidney (15). An alternative for the Wnt pathway activation is to inhibit GSK3 (Glycogen Synthase Kinase 3) using CHIR99021 (15–18). The organization of the posterior mesoderm into paraxial, intermediate and lateral mesoderm can be controlled by combinations of BMP4, Activine/Nodal, FGF9 (Fibroblast Growth Factor-9) (15) or BMP7/CHIR99021 (19) or FGF2/Retinoic acid (RA) (20). Various approaches can be used to specify the intermediate mesoderm into GATA3+ cells (anterior MI and BU cells) including FGF9 and RA. Thus, different renal cell types could be generated from human PSCs such as renal progenitors (21) IM cells capable of forming proximal tubular cells (20) and UB-like cells capable of integrating into mouse kidney embryos while contributing to their development (22).

In order to mimic the complexity of the kidney as a whole, these protocols have shown their limitations. Thus, efforts have focused on the generation of organoids by applying technologies that already work on NPCs, i.e., 3-dimensional culture, and the first renal organoids from pluripotent stem cells have been reported in 2014. Taguchi et al. have described the differentiation of PSCs into MM capable of organizing themselves into renal structures including glomeruli with podocytes and renal tubules (23). Subsequently, Morizane et al. produced organoids containing tubules with segmentation, which self-organize into pseudo-glomerular structures with the presence of proximal tubules, Henle loop and distal tubules (17). Finally, Freedman et al. yielded organoids containing renal tubules, podocytes and endothelial cells (16).

To summarize as closely as possible the structure of a kidney and all its components, it was necessary to generate both MM-derived and UB-derived cells. These conditions were satisfied in the study of Little's group, which developed a protocol to induce the 2 types of populations from human embryonic stem cells, using a sequence based on CHIR99021 and FGF9 to generate both MM and UB cells, via the primitive streak and IM in 7 days of differentiation. At this stage, the cells are detached and then cultured on a Transwell filter in order to place the cell suspension at a favorable culture medium/air interface for the cells' self-organization (15). The resulting structures notably contain collecting duct cells derived from the UB but also proximal-tubule like cells derived from MM, showing that an interaction between the cell types has actually occurred in similar ways as *in utero* kidney development.

From that time, the protocol has been improved to produce similar proportions of UB and MM cells, leading to remarkably organized organoids. We note the presence of “nephron-like” structures, glomeruli, proximal tubules, Henle loop and cells from collecting tubes, strongly reproducing the organization of a human kidney, with stromal cells and a vascular network containing capillaries. Transcriptomic analysis shows a gene expression profile similar to that of human embryonic kidneys (18).

In terms of functionality, renal organoids have a selective endocytosis capacity of “dextran cargoes” and respond to nephrotoxic agents (16, 18).

### Improving the Differentiation, Maturation, and Characterization of Kidney Organoids

Despite spectacular progresses in the field, one of the first limits in the generation of kidney organoid is the variability among differentiation protocols. Indeed, whatever the targeted lineage, differentiation protocols are subjected to both batch-to-batch and line-to-line variations implying that protocols often have to be adapted for each cell line of interest. Regarding kidney organoids, Phipson et al. showed that the main source of transcriptional variation in a specific kidney organoid protocol appeared between experimental batches (and not iPSC clones) especially for genes linked with temporal maturation. Indeed, distinct iPSC clones showed congruent transcriptional programs. Observed interexperimental and interclonal variations were strongly associated with nephron patterning (24).

Regarding kidney organoid characterization, studies based on RNA sequencing (single cell RNA seq and single nucleus RNA seq) suggest that kidney organoids differentiated using two different published protocols show more commonalities than differences (25). Importantly, despite expected cell types within the organoids, some off-target cell types have been highlighted, including neurons, and muscle cells. Overall, these RNAseq studies converge showing the absence of collecting ducts and the general immaturity of the organoid cell types, compared with either fetal or adult human kidney cells (26, 27).

Thus, maturation of the generated structures is a crucial point. In this field, technical improvements have been performed by fine tuning the extracellular environment. Garreta et al. developed a transplantation strategy that uses a chick chorioallantoic membrane to promote implanted organoid growth and differentiation as well as a vascular component. They reproduce this finding *in vitro* manufacturing hydrogels with a compound close of chorioallantoic membrane which accelerates organoid differentiation, generating kidney organoids that transcriptomically matched second-trimester human fetal kidneys (28).

With the same ambition of better mimicking *in situ* conditions, Homan et al. placed partially differentiated kidney organoids onto an adherent layer of extracellular matrix housed within a 3D printed millifluidic chip. Application of flow over the organoid surface led to enhanced differentiation and expansion of the endogenous endothelial cell population, forming robust vascular network that can anastomose between adjacent organoids. Moreover, enhanced polarization and maturation or epithelial and glomerular regions have been observed, compared to static culture conditions (29).

High throughput techniques have also been applied to renal organoids from PSCs. Czerniecki et al. developed an automated High-Troughput-Screening (HTS) platform to improve the differentiation and phenotyping of human renal organoids. They carried out the entire differentiation protocol (21 days) automatically on cell culture robots. The High-Content-Screening analyses revealed a dose-dependence and threshold effect of the compounds used during differentiation, and they were also able to highlight differentiated compartments that were not previously identified, with the presence of interstitial and parietal cells. Finally, they performed chemical screening to assess nephrotoxicity on renal organoids, including an unexpected role of myosin in renal polycystosis using differentiated renal organoids from iPSC modified by genome editing (27). One of the barriers remains to generate enough renal organoids for these applications. In this sense, Przepiorski et al. have developed an effective protocol for scale up generating renal organoids at limited cost using a bioreactor (30).

In addition, with recent advances in the field of bioprinting, two studies reported the use or roboting plating system to enable to production of large numbers of highly reproducible organoids, showing equivalent morphology, component cell types and gene expression compared with structures generated manually. Using bioprinting will for sure accelerate the use of kidney organoids in pharmacology and toxicology studies (31, 32).

Of note, a “glomerular chip” or organoid glomeruli containing only mature podocytes derived from iPSC has also been developed, combining organ-on-chip microfluidic techniques with specific differentiation protocols. These *in vitro* chips structurally and functionally mimics the glomerular membrane (33, 34).

Finally, other structural adaptations include organoids being incorporated in “organ-on-a-chip,” which are devices including a microfluidic system of small channels that are used to provide nutrients to the cells inside. By using cells grown in organoid cultures to populate the organ-on-a-chip system, researchers add a level of biological relevance and complexity not achievable with either system alone; opening the door to the possibility to also connect on one chip different organoids (kidney and bladder organoids, for example) to generate more complex systems.

## Combining Kidney Organoids With Genome Editing

Like iPSCs in 2006, a major breakthrough revolutionized the world of cell and molecular biology in 2012: genome editing techniques, a modular system for cleaving a DNA sequence at a targeted location using a bacterial endonuclease “programmable” by a specifically selected RNA (RNA guide). This precision technology called CRISPR (Clustered Regularly Interspaced Short Palindromic Repeat) has since shown its effectiveness in human cells (35), and has been rapidly applied to iPSCs, allowing in particular the modeling of genetic diseases without having to use iPSCs derived from patients' cells. This section will present the main studies which combine the use of genome editing with kidney organoid technology.

### Better Understanding Human Kidney Development and Regeneration

Firstly, organoid and gene-editing technologies can be combined to interrogate and dissect human lineage relationships *in vitro*. Using mouse embryonic stem cells deleted for Wnt4 by CRISPR/Cas 9 systems, Tan et al. showed that cells from the metanephric mesenchyme of Wnt4-deficient organoids depict to undergo the MET which normally occurred in response to the signaling from the ureteric bud, whereas MET was maintained in WT organoids (36). Similarly, Howden et al. used CRISPR/Cas9 gene-edited lineage reporter lines to show that, within the organoid, SIX2-expressing cells give rise to nephron epithelial cells but not to ureteric epithelium. However, at the contrary to what was observed on mice models (37, 38), authors fail to show the presence of a nephron progenitor niche within the organoid capable of self-renewal and nephrogenesis (39).

In addition, CRISPR technology has been used to develop a system in which renal differentiation, glomerular maturation, and podocyte phenotype can be evaluated within organoids by fluorescence microscopy using reporter genes and the extinction of NPHS expression (40).

Kidney organoids also represent an interesting model for understanding mechanisms involved in the development of kidney damage associated with intense stress. Indeed, we have observed in the laboratory that many signaling pathways and genes activated during embryonic development of the kidney and therefore in the generation of renal organoids are reactivated in a mature kidney that has undergone major ischemic injury (41). A cold ischemia sequence in a mature pig kidney induces the activation of the RNA polymerase-associated protein LEO1 gene, which is a component of the polymerase-associated factor 1 complex (PAF1C) required for the transcription of the Wnt, Hox and Notch genes, and involved in the development and maintenance of embryonic stem cell pluripotency (42). Ischemic lesions, in the same porcine model, induce the under-expression of the RHOU (Ras Homolog Family Member U) gene regulated by a Wnt-induced gene. RHOU in conjunction with Wrch (Wnt-1 responsive CDC42 homolog) is involved early in the development of multicellular organisms. Similarly, porcine kidneys that have undergone ischemic lesions have an under expression of CDC42 homolog (Cell division control protein-42 homolog) which plays an essential role in survival, growth, and development (41).

### Disease Modeling and Drug Screening

Disease modeling with iPS-derived renal cells is possible without the use of genome editing. Indeed, disease specific models have been generated by selecting the desired genotype for iPSC generation. Podocytes differentiated from iPSCs from patients suffering from Alport syndrome exhibit dysfunctional integrin signaling and potassium channel function (which may be associated with podocyte loss) (43). Glomerular chips generated from iPSCs from a patient with congenital nephrotic syndrome (mutation NPHS1 mutation) displayed reduced level of podocin and nephrin (33).

Using CRISPR/Cas9 to specifically induce one mutation is the other option to model specific genetic disease, allowing also the precise control of the genetic background. The first CRISPR-generated genetic model of renal organoids modeled polycystosis (PKR), the most common renal genetic disease. The disease is characterized by the development of renal cysts and fibrotic lesions, deteriorating renal function. In its dominant form, PKR is induced by mutations in the PKD1 and/or PKD2 genes encoding fibrocystins 1 and 2, respectively. From human iPSCs, losses of function by genetic mutation have been induced either on the PKD1 gene or on PKD2 by CRISPR. These mutations did not affect the ability of iPSCs to differentiate into renal organoids. However, a few days after the end of differentiation, a small but detectable proportion of organoids developed cysts, a phenomenon that was not observed for isogenic control lines (the same iPSCs line but without mutation induction). This study shows that cyst formation is an intrinsic phenomenon of mutated cells and can therefore be reproduced *in vitro* (16).

Subsequently, it was observed by video microscopy that cysts were formed from whole tubular structures that partially detach from the culture box; using low adhesion culture plates, authors observed a very high cyst formation rate for mutated organoids, which was significantly higher than previous culture conditions, while this rate remained low for iPSC control lines. This observation highlights the strong tendency of epithelial cells to form cysts under non-adherent 3-dimensional condition, and shows that the PKD mutation has a major role to in promoting cystogenesis in renal organoids. The produced cysts have proximal, distal and proliferation markers, which is a feature of cysts obtained from biopsies of patients with PKD. This work shows the critical role of the cell environment and adhesion forces for cystogenesis (44). It is possible to culture renal cells from biopsy of patients with autosomal dominant PKR (45) but there is heterogeneity depending on the cellular sources and epigenetic background of the patients. The renal organoid model derived from iPS therefore offers a significant advantage that of being able to compare the cells generated with isogenic control iPSCs lines, without having to consider possible variations in epigenetic profile or interpatient differentiation efficiency.

In addition to disease modeling, renal organoids can also be used to identify new targets. Podocalyxin (PODXL1) is strongly expressed in podocytes and is therefore a candidate gene for segmental and focal glomerulosclerosis (FSGS), a complex, rare, heterogeneous, and poorly understood disease characterized by various histological lesions leading to a functional defect of the glomerular filtration barrier. In culture, primary podocytes do not proliferate and rapidly de-differentiate. This de-differentiation is associated with loss of podocyte feet and extinction of nephrine expression. It is possible to specifically differentiate human iPSCs into a homogeneous population of podocytes which, once transplanted into mice, mature at the glomerulus level, and are vascularized (46). Renal organoids may also contain podocytes but their state of maturation was not known until a study showing that they are similar to podocytes present in the *in situ* kidneys in terms of gene expression and ultrastructure with the progressive formation of junction-rich basal membranes and microvilli-rich apical membranes. Using deleted iPSCs for the PODXL gene, Kim et al. generated organoids whose podocytes show a defect in the microvilli assembly with spaces between cells resulting in porous junctions. These defects were already identified in murine models of this disease, showing that podocalyxin has an essential and preserved role in the maturation of podocytes and the development of FSGS in particular (47).

### Phenotypic Correction

Correcting abnormal phenotype by directly targeting causal mutation is one of the promising application of precise gene editing. Little's team has used renal organoids as a platform for functional validation of new genetic variants potentially implicated in the development of kidney diseases. By high-throughput sequencing, they demonstrated a potential causal mutation on the IFT140 gene in a proband individual with nephronophilia. This gene codes for a subunit of the intraflagellar transport complex A. The organoids carrying this variant have shortened tubules and morphologically abnormal cilia. iPSCS corrected for this candidate mutation were generated by CIRSPR, and the gene correction allowed to reverse the abnormal phenotype. Transcriptomic analyses in epithelial cells isolated from these organoids show down-regulation of genes associated with apico-basal polarity and cell junctions and this polarization defect has been confirmed in matrigel cyst formation assays (48).

Tanigawa et al. generated hiPSCs from a patient with an NPHS1 misense mutation and differentiated them into organoids. They observed that induced podocytes exhibit impaired NEPHRIN localization which is supposed to be present at the cell surface playing a major role in the slit diagram formation process and therefore the glomerular filtration barrier. Once injected into immunodeficient mice, mutant podocytes developed foot process but did not form slit diagram. Using CRISPR/Cas9 to correct the single amino acid mutation, authors restored NEPHRIN localization and colocalization with other proteins from the slit diagram and therefore slit diagram formation (49).

## Conclusion

Reproducing kidney structure *in vitro* became a realistic possibility: these organoids grown in the laboratory can be used to understand biological mechanisms, develop drugs, research new therapies, or even for personalized medicine. This innovation revolutionizes research by offering a fast alternative (differentiation of iPSCs in less than a month, saving time compared to embryogenesis and organ maturation in animals), at limited cost since compatible with high-throughput applications. The development of this technology also makes sense where the use of animal models for research purposes should be reduced to a minimum. Inter-organ interactions still need to be taken into account; in this field too, progress are dazzling, and technologies based on body-on-chip microfluidics are emerging, suggesting the possibility of connecting renal organoids to other organoid structures (liver, bladder) using physiological fluids and partially reproducing *in vitro* the complexity of living systems at the cellular, intercellular, intertissular, and inter-organ levels. This field will continue to expand based on new and more consistent methodologies (50). In addition, combining the results from other technologies with those from organoids will also provide finer-grained understanding of biological systems and open new pathways to disease treatments.

## Author Contributions

CS, SG, and TH wrote the manuscript and approved its final version.

### Conflict of Interest

The authors declare that the research was conducted in the absence of any commercial or financial relationships that could be construed as a potential conflict of interest.
